# Differences in motivation and adherence to a prescribed assignment after face-to-face and online psychoeducation: an experimental study

**DOI:** 10.1186/s40359-017-0172-5

**Published:** 2017-01-26

**Authors:** Sven Alfonsson, Karin Johansson, Jonas Uddling, Timo Hursti

**Affiliations:** 1 Department of Public Health and Caring Sciences, Uppsala University, Box 564, 751 22 Uppsala, Sweden; 2 Centre for Psychiatry Research, Department of Clinical Neuroscience Karolinska Institutet & Stockholm Health Care Services, Stockholm County Council, Sweden; 3 Department of Psychology, Uppsala University, von Kraemers allé, Box 1225, 751 42 Uppsala, Sweden

**Keywords:** Adherence, Motivation, Psychoeducation, Internet, Homework assignments

## Abstract

**Background:**

Adherence to treatment homework is associated with positive outcomes in behavioral psychotherapy but compliance to assignments is still often moderate. Whether adherence can be predicted by different types of motivation for the task and whether motivation plays different roles in face-to-face compared to online psychotherapy is unknown. If models of motivation, such as Self-determination theory, can be used to predict patients’ behavior, it may facilitate further research into homework promotion. The aims of this study were, therefore, to investigate whether motivation variables could predict adherence to a prescribed assignment in face-to-face and online interventions using a psychotherapy analog model.

**Methods:**

A total of 100 participants were included in this study and randomized to either a face-to-face or online intervention. Participants in both groups received a psychoeducation session and were given an assignment for the subsequent week. The main outcome measurements were self-reported motivation and adherence to the assignment.

**Results:**

Participant in the face-to-face condition reported significantly higher levels of motivation and showed higher levels of adherence compared to participants in the online condition. Adherence to the assignment was positively associated with intrinsic motivation and intervention credibility in the whole sample and especially in the online group.

**Conclusions:**

This study shows that intrinsic motivation and intervention credibility are strong predictors of adherence to assignments, especially in online interventions. The results indicate that intrinsic motivation may be partly substituted with face-to-face contact with a therapist. It may also be possible to identify patients with low motivation in online interventions who are at risk of dropping out. Methods for making online interventions more intrinsically motivating without increasing external pressure are needed.

**Trial registration:**

clinicaltrials.gov NCT02895308. Retrospectively registered 30 August 2016.

## Background

Homework assignments is one of the essential components in effective behavioral psychotherapy since it is associated with positive treatment outcomes and, in contrast to many other variables, may be affected by treatment design and therapist behavior [1, 2]. However, adherence to assignments is often only moderate, and patients report obstacles such as time restraints and competing priorities [3]. It is, therefore, important to investigate factors, such as motivation, that are associated with adherence to prescribed assignments in more detail [4]. Completing assignments, such as reading texts and doing exposure exercises, is not typically naturally reinforcing for patients and thus a behavior that is hard to initiate and maintain [5].

Therapists may act as “reinforcement machines” and provide positive attention, praise and encouragement for patients’ efforts to complete homework [6]. They can also clarify and highlight that complying with assignments are in line with the long-term goals of the patient [5]. Therapists hold patients accountable for completing homework and patients are probably mildly negatively reinforced for adhering to assignments if they expect the therapist to follow up on homework [7, 8]. There may be reasons to investigate patients’ perceptions more closely since behavior that is intrinsically reinforced is, for example, more durable than extrinsically reinforced behavior [9, 10]. The different processes and effects on internal and external motivation have been investigated in studies on homework assignments in psychotherapy [11]. In previous studies, Kazantzis and colleagues have identified that patients that feel engaged in the treatment and receive positive feedback are more adherent to homework. They have further provided a therapist checklist and an instrument to measure patients’ experience of assignments, the Homework Rating Scale II (HRS II) [12]. However, there is still a need to better understand the processes behind homework adherence in order to improve clinical outcomes [13].

One model that can be used to describe how different types of operant contingencies affect health behavior is Self-determination theory (SDT) [14, 15]. In this model, the term *motivation* is used to describe the conscious reason for a behavior rather than the operant function, which means that it refers to the antecedent reason or expectation of a behavior rather than the consequences. The primary focus of the model is to differentiate between different sources of motivation and the degree to which they are internalized [16]. The model describes five types of motivation that are divided into two groups: the intrinsic-, identified- and integrated types of motivations are called autonomous (i.e., internal) motivation while external- and introjected types of motivations are called externally regulated motivation. Depending on the type of motivation, different effects on health behaviors and school work have been observed [15]. For example, people who report autonomous motivation are more likely to succeed in maintaining health behaviors such as smoking cessation, arguably because they are less dependent on external factors [17]. Even though motivation often originates from external sources, SDT states that the process of internalizing motivation for functional behaviors, i.e. going from controlled to autonomous motivation, is an important factor in explaining the maintenance of behavior [18, 19]. In psychotherapy, psychoeducation is used to clarify the rationale for behavior change which should result in the patient doing assignments of her own free will. According to SDT, this process consists of going from external to autonomous motivation for a new behavior [20]. Previous researchers have suggested that that psychotherapy working alliance, a central construct in psychotherapy research, is best conceptualized in Cognitive Behavior Therapy (CBT) as a process of collaborative empiricism between therapist and patient [21]. According to this view, therapists should avoid using external pressure on patients and not provide answers but rather use guided discovery to help patients become less reliant on external stimuli and consequences and instead focus on drawing their own conclusions about their thoughts, feeling and behavior. This strategy seems to be beneficiary for patients and could be understood as an example of internalizing motivation in the SDT theoretical framework.

Compared to other theories of motivation such as the Theory of Planned Behavior [18], SDT focuses on both the different types of motivation and the process of how motivation transform and change depending on external factors. While the different theories of motivation are largely concordant, SDT is easy to use in conjunction with operant principles to investigate and understand the process when therapists work to motivate patients and the patients’ subsequent adherence to psychotherapy homework [16]. If assignments are perceived as interesting and consistent with long-term goals, they will be intrinsically positively reinforced and such autonomous motivation will facilitate behavior change [22]. Previous studies have shown that increasing treatment motivation using Motivational Interviewing before treatment start may improve treatment adherence and outcomes, especially for patients with high symptom levels [23–25]. Extrinsic positive reinforcement, such as the therapist’s praise, may compensate intrinsic motivation for difficult or unpleasant assignments such as exposure exercises [23]. Also, if patients perceive that they are accountable for completing assignments this behavior may be extrinsically negatively reinforced, or externally regulated, which may also facilitate behavior change. There is a delicate balance for therapists using external control for fostering homework adherence and studies have shown that homework adherence and treatment outcomes are both associated with therapist skill [26]. Such accountability arguably depends on personal contact with a therapist and this may therefore partly explain why guided (i.e., therapist-aided) psychotherapy is often more effective than self-help in both face-to-face and internet-based contexts [27–29].

Internet-based psychotherapy is a valuable alternative to face-to-face treatment but the levels of adherence may be marginally but significantly lower than in traditional therapy, even in online treatments that include contact with a therapist [30, 31]. Therapist support seems to be the most important factor affecting adherence in online psychotherapy, but the reasons have not been studied in detail [32]. For example, working alliance in online therapy seems to be on par with that of face-to-face psychotherapy, but there may be important differences in the deliverance and perception of human support between the two modalities [33]. Whether therapist support primarily acts as encouragement and other forms of positive reinforcement, as external pressure to foster accountability or a mixture of both is still unclear [34]. In both face-to-face and online psychotherapy, patient adherence to the treatment program, including completing assignments, is one of the best predictors of treatment outcome [35]. In order to design more effective interventions, it is important to better understand what factors affect patients’ adherence to online treatment [36]. Whether such differences in how therapist support is perceived and how it affects intrinsic and extrinsic motivation for assignments in face-to-face and online therapy has not been studied. While therapist support may affect adherence to assignments during a treatment, it has also been found that initial treatment credibility is an important factor for treatment adherence and outcome, but the exact mechanisms are as yet unclear [37]. There is thus a need for more experimental studies on factors such as support, motivation, and credibility that may affect treatment adherence as well as the mechanisms behind these effects. A better understanding of how different reinforcement can be used in psychotherapy may lead to improved treatments and in the end better help for more patients.

In conclusion, patients’ adherence to assignments is affected by both autonomous and externally regulated motivation. Therapist support via the Internet may provide a weaker social bond and result in lower levels of externally regulated motivation. It may be that Internet-based psychotherapy relies on patients having autonomous motivation and since studies using self-referral may attract such individuals, it may result in attrition rates that are similar to that of face-to-face psychotherapy [38]. Whether different types of motivation have a different impact on adherence in face-to-face and online psychotherapy is however largely unknown.

The aims of this study were to investigate (1) participants’ autonomous and externally regulated types of motivations to complete a typical psychotherapy assignment, (2) participants’ subsequent adherence to the prescribed assignment and the associations between autonomous and externally regulated motivations on the one hand and adherence on the other and (3) any differences regarding types of motivations, adherence and their associations between the face-to-face and online conditions.

The hypotheses were (1) that participants would report higher autonomous motivation than externally regulated motivation, (2) that autonomous motivation and externally regulated motivation would be positively associated with adherence, (3) that participants in the face-to-face condition would report higher autonomous motivation and lower externally regulated motivation as well as higher adherence to the assignments compared to participants in the online condition.

## Methods

To investigate the association between motivation and adherence to assignments in face-to-face and online settings, this study had a longitudinal randomized design with two conditions. The two conditions were face-to-face psychoeducation with a therapist and online psychoeducation with therapist support. A psychotherapy analog model with a one-session intervention for a non-clinical population was used. Data was collected at baseline and at seven to nine days follow-up. The study was designed following the CONSORT guidelines for clinical trials.

### Participants and procedure

Participants were recruited by advertisement at a university campus among people who showed an interest in better understanding their every-day behaviors and well-being. Potential participants were informed about the study and those showing interest were asked to fill out a contact form. Each person was subsequently contacted by telephone and was provided further information about the study, including the fact that the intervention did not comprise a treatment. They were presented with a description of the study procedure and invited to ask questions. They were also evaluated regarding the inclusion and exclusion criteria and had an opportunity to ask questions. The inclusion criterion was having at least one problematic behavior one wished to understand or change. Exclusion criteria were being below 18 years of age, having no access to a mobile phone and the Internet, reporting elevated levels of depressive symptoms according to the screening instrument (see below) or currently attending psychotherapy. Those who chose to participate were asked to complete the background and screening instruments before being randomized to either of the two conditions using a random number list obtained from https://www.randomizer.org/. Participants who reported elevated symptoms of depression on the screening instrument were contacted and referred to standard care. All participants were followed up after study end to provide feedback on the study.

Participants in the face-to-face condition met with a therapist and received a 30–40 min psychoeducation. After the psychoeducation, they were asked to complete instruments regarding their motivation for the prescribed assignment. These instruments were completed without the therapist present in the room and participants were asked to put them in a sealed envelope only marked with their participant code number in order to minimize social pressure bias.

Participants in the online condition were given log in information for the web page and if they had not logged in within two days, were reminded by e-mail and text message to do so. A total of two such reminders were sent if necessary. After having completed the online psychoeducation, participants were asked to complete instruments about their motivations for the assignment. They thereafter had complete access to the web page and could access the psychoeducation and the assignment form as often as they needed during the following nine days.

### Intervention

The intervention consisted of a psychoeducation component taken from affect focused psychotherapy as described by McCullough and Magill [39]. In this model, emotions are physiological patterns that are shaped mainly in the context of previous relations. By using the model, patients are helped to better understand their current emotions, behaviors, and cognitions. The aim of the intervention used in this study was to provide information about the six basic affects and how they may influence everyday behaviors and well-being in recurring patterns. The psychoeducation included two case vignettes and prompted the participants to fill out their own examples of emotional situations they had experienced. The presentation concluded with an assignment that instructed each participant to record six previous situations in which they had experienced an emotion that affected their behavior or well-being and also to register and analyze one emotional situation each day the coming week. In total, each participant was thus asked to register and analyze 13 emotional reactions. This procedure was designed to mimic the way the affect model can be used in psychotherapy and also to be an analog to how assignments in Cognitive Behavioral Therapy, such as recording negative automatic thoughts, are typically designed. Further, psychoeducation has shown to have a small but significant effect on symptoms of psychological distress, even when offered as a stand-alone intervention [40]. It is, therefore, possible that even a short but theoretically sound intervention, such as the one used in this study, may have some effect on well-being and thus feel relevant for participants.

After the psychoeducation, participants in both groups had access to a secure web page with the standardized registration form for the assignment. They could log in and fill out the form as often as they wished and could for example complete one part of the assignment per day of the study or complete all parts of the assignment at one occasion. The web page automatically saved all input data so participants could fill out some of the assignments and then later log in to complete the rest at a later time. In both conditions, participants had a maximum of 9 days to complete the assignments and all received an automatic e-mail reminder after 7 days. This procedure for registering an assignment is typical for internet-based psychotherapy but deviates from the typical procedure used in standard in vivo psychotherapy which often uses paper forms. However, the same online procedure was used in both conditions of this study in order to remove the potential effect of using online data collection in only one group and the increased risk of missing data that was expected from providing participants with paper forms.

### Conditions

In the face-to-face condition, the psychoeducation was provided by one senior psychologist and two psychology master students. The intervention was manualized and the therapists met and discussed and role-played their presentations in order to ensure adequate reliability. Each therapist was instructed to follow a written manuscript but was allowed to check in with participants, to ask questions, to use idiosyncratic examples and to provide feedback. They were not allowed to stray from the manuscript or to provide information or content that was not covered. In the face-to-face condition, no online material was used. The psychoeducation took approximately 30–40 min for each participant.

In the online condition, the same written manuscript for psychoeducation as in the face-to-face condition was used. This material was presented both as a video presentation as well as text on the webpage. The same examples as in the face-to-face condition were used and participants were asked to submit their own examples where appropriate. The intervention content for the online condition consisted of four items: a video presentation, a text, two case vignettes and a complete assignment example that could be accessed in any order. There was also an online therapist who greeted each participant the first time they logged in and was available to answer any questions and provide feedback. The online therapist spent approximately 5–10 min per participant in this study which was spent on writing welcome messages and answering questions. All communication between participants and the online therapist was asynchronous. Participants in the online condition had full access to the web page content and online therapist during the course of the study.

The two conditions thus included the same intervention and only the format of presentation, orally in the face-to-face condition and through text and video material in the online condition, was different. Both conditions used the same web page for registering the assignment and all participants received e-mails with the same reminders for completing the homework and study instruments.

### Measurements

The outcome variables of this study included five measurements of adherence: First, whether a participant started the intervention as agreed after the telephone assessment was measured dichotomously (yes/no). For participants in the face-to-face condition, showing up and participating in the psychoeducation appointment was considered having started the intervention. For participants in the online condition, logging into the web page and accessing any of the intervention content was considered having started the intervention. Second, the total number of log in occasions for working on the assignment (i.e., after accessing the intervention) was measured. Third, whether a participant subsequently completed any part of the assignment was also measured dichotomously (yes/no). Fourth, the total time spent on the web page was logged for each participant at study end. Fifth, the number of prescribed assignments that each participant had completed on the web page form was measured. This variable ranged from 0 (not completed any assignment) to 13 (completed all assignments).

Motivation for the assignment was measured with the Situational Motivation Scale (SIMS). The SIMS was developed based on the Self-determination theory to measure motivation in experimental tasks [41]. The SIMS comprises 16 items on four subscales, Intrinsic motivation (e.g., “I think that this activity is interesting”), Identified regulation (e.g., “I am doing it for my own good”), External regulation (e.g., “I am supposed to do it”) and Amotivation (e.g., “I don’t see what this activity brings me”), corresponding to the analogue constructs described in SDT. The SIMS contains 4 items per subscale scored on a scale from 1 to 7 providing a score between 4 and 28 for each subscale. It has been mainly used in sport- and health psychology and shown adequate psychometric properties [42]. In this study, the internal reliability was α = .74 - .83 for the four subscales.

Since intervention credibility has shown to be an important factor in predicting psychotherapy adherence, the Treatment Credibility Scale (TCS) was also used in this study [43, 44]. The TCS comprise five items scored on a scale between 1 and 10 providing a total score between 5 and 50. The TCS has been widely used in internet psychotherapy research, but its psychometric properties are largely unknown. In this study, the internal reliability was α = .86.

In order to explore the factors suggested by Kazantzis [11], the SIMS was complemented with Visual Analogue Scales (VAS) created for this study based on the Homework Rating Scale. The HRS II is designed to be used during psychotherapy and in collaboration between therapist and patient in order to explore and improve homework engagement. The reasons for not using the HRS II in this study was that three of the items of the HRS II specifically refer to ongoing therapy and that the HRS II does not measure personal bond between therapist and patient, a factor that is probably important for homework adherence. Instead, the VAS-scales were designed to measure the relevant constructs included in the HRS II but adapted to the experimental intervention format used in this study and included a factor for therapeutic bond, resulting in six constructs: therapist expertise and benevolence, accountability, sense of pleasure and mastery, relevance, encouragement and collaboration, and obstacles. The Expertise and benevolence scale was conceptualized as therapist expertise, therapist effort, therapist benevolence, therapist friendliness and trust in the therapist. The Expertise and benevolence scale was conceptualized as participants’ perception of the therapist as knowledgeable, trustworthy, benevolent, friendly and making an effort. The Accountability scale was conceptualized as participants’ self-rated responsibility, feelings of guilt, a perception of being monitored, feelings of embarrassment for not completing the assignment and negative expectancies. The Sense of pleasure and mastery scale was conceptualized as expectations of experiencing interest, personal development, meaningfulness, pleasantness and appreciation from working with the assignment. The Relevance scale was conceptualized as the expected ability of the intervention to be helpful, to lead to better self-understanding, its importance, being an interesting experience and lead to personal development. The Encouragement and collaboration scale was conceptualized as experiencing encouragement, practical support, constructive feedback, praise and appreciation from the study staff. The Obstacles scale was conceptualized as the perceived burden or cost of the working with the intervention, including time, frustration, unpleasantness, complexity and practical difficulties. Each VAS-scale had five items scored between 0 (not at all) and 100 (completely) resulting in a mean score between 0 and 100 for each construct as well as an index for the whole instrument. These VAS-scales were designed for this study, and the psychometric properties are therefore unknown but in the current study, the internal reliabilities were α = .71 – 93 for the six subscales.

To screen for depressive symptoms among participants, the short version of the Depression, Anxiety and Stress Scale (DASS) was used [45]. The DASS contain 21 items and three subscales; Depression, Anxiety, and Stress. Each subscale ranges from 0 to 21 and a cutoff of 11 on the Depression subscale was used to identify elevated symptoms. The DASS has shown adequate psychometric properties in previous studies [46]. The internal alpha scores in this study were Depression = .86, Anxiety = .71 and Stress = .84 for each subscale respectively.

Background variables, age, gender, marital status and previous experience of psychotherapy were collected from each participant at inclusion. Study feedback was obtained by contacting each participant by e-mail at study end.

### Analyses

The normality of data distribution was investigated prior to analyses and several variables were found to be skewed. Since the transformation of data did not improve distributions substantially, it was decided to use non-parametric statistical testing of group differences and forego regression analyses for prediction. Instead, the associations between background variables age, gender and marital status, the SIMS and the VAS-scales on the one hand and the outcome variables on the other hand were investigated using non-parametric correlation analyses (Spearman’s *rho*). Some of the VAS-scales were expected to be inter-correlated but unfortunately, there is no feasible non-parametric method for analyzing the unique variance in multivariate data. Instead, correction for multiple comparisons of associated variables was calculated with intercorrelations of r = .5 providing an adjusted p-value threshold of .01 [47]. Also, the VAS-scales Index was included as the general measure of homework engagement.

Differences in variables and between study conditions were analyzed with Wilcoxon Signed Rank Tests, *Chi* ^2^, and Mann–Whitney tests. *r* as was used as a measure of effect size with *r* = .1 equals small, *r* = .3 equals medium and *r* = .5 equals large effect sizes. A *p*-value of .05 was considered the threshold for statistical significance in all analyses. In order to find correlations with small effect sizes using a .05 significance level and .80 power, 80 participants were needed to be included in this study. To allow for dropout and missing data, it was decided that a total of 100 participants should be included in the study. Missing values (*n* < 1%) were imputed using Expectation-Maximization procedures.

## Results

**Fig. 1:**
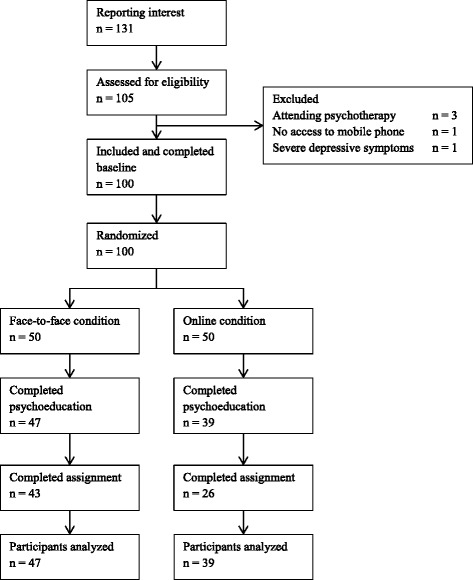
CONSORT flow chart

A total of 131 persons showed interest in the study and 105 were contacted by telephone, see Fig. 1. Of these, three were excluded due to currently attending psychotherapy, one was excluded for not having access to mobile phone and the Internet and one was excluded due to reporting depressive symptoms and being referred to standard care. A total of 100 people were included in this study with 50 randomized to each condition. Of these, all were university students, 68 (68%) were women, 55 (55%) were cohabitant, 45 (45%) were single and 8 (8%) had previously had psychological treatment. The mean age was 24.9 (*SD* = 7.1) years. The mean values and standard deviations for the DASS subscales were Depression = 4.2 (3.6), Anxiety = 2.7 (2.5) and Stress = 6.6 (4.2). There were no significant differences between the conditions regarding any variables at baseline.

### Motivations

After the intervention but before starting the assignment, participants scored significantly higher on the SIMS Intrinsic (*Z* = 6.27, *p* < .001, *r* = .67) and Identified (*Z* = 6.28, *p* < .001, *r* = .68) compared to the Extrinsic subscale. Participants in the face-to-face condition scored significantly higher on the SIMS Intrinsic subscale (*Z* = 4.50, *p* = .001, *r* = .49) and the TCS (*Z* = 5.19, *p* = .001, *r* = .57) and significantly lower on the SIMS Amotivation subscale (*Z* = 2.04, *p* = .042, *r* = .22) compared to participants in the online condition. On the complementary VAS-scales, participants in the face-to-face condition scored significantly higher on the Expertise and benevolence (*Z* = 3.02, *p* = .003, *r* = .33), Pleasure and mastery (*Z* = 2.07, *p* = .041, *r* = .23), Encouragement (*Z* = 2.77, *p* = .006, *r* = .30) scales as well as lower on the Obstacles (*Z* = 2.17, *p* = .039, *r* = .24) scale compared to participants in the online condition. The results from the self-report instruments and the differences between the groups can be seen in Table 1.

**Table 1:** Results from the self-reported instruments after the intervention but before starting the assignment (*n* = 86)

Measurement	All *M* (*SD*)	Face-to-face *M* (*SD*)	Online *M* (*SD*)	*Z*	*p*	*r*
SIMS Intrinsic	16.16 (4.6)	17.9 (3.5)	13.9 (5.0)	4.50	.001	.49
SIMS Identified	19.1 (4.7)	18.9 (5.4)	19.4 (3.7)	0.59	.554	.06
SIMS Extrinsic	6.2 (2.3)	6.2 (2.3)	5.8 (2.7)	0.71	.475	.08
SIMS Amotivation	7.0 (2.9)	6.7 (3.2)	7.4 (2.4)	2.04	.042	.22
TCS	33.1 (6.6)	36.0 (6.2)	29.3 (4.9)	5.19	.001	.57
Expertise and benevolence	79.0 (11.4)	84.2 (9.8)	68.8 (13.7)	3.02	.003	.33
Accountability	65.2 (15.4)	68.1 (17.1)	57.7 (15.9)	1.50	.135	.16
Pleasure and mastery	67.3 (16.8)	72.0 (16.5)	55.2 (20.8)	2.07	.041	.23
Relevance	65.3 (19.1)	66.0 (24.6)	57.5 (19.7)	1.16	.247	.13
Encouragement	54.6 (14.6)	59.0 (16.8)	44.8 (14.4)	2.77	.006	.30
Obstacles	34.0 (13.6)	29.9 (13.7)	39.5 (11.6)	2.17	.039	.24
Index	65.5 (12.1)	70.6 (12.0)	58.9 (12.0)	4.53	<.001	.49

Note. *SIMS* situational intrinsic motivation scale, *TCS* treatment credibility scale

### Adherence

The number of participants who dropped out from the study before completing the psychoeducation was significantly higher (*χ* ^2^ = 5.32, *p* = .021) in the online condition (*n* = 11, 22%) than in the face-to-face condition (*n* = 3, 6%). In the whole sample, participants logged in a mean number of 4.6 times during the intervention and they spent a mean number of 89.2 (*SD* = 85.0) minutes on the web page, i.e. about 1.5 h. Participants in the face-to-face condition had significantly more log in occasions to fill out the assignment form (Z = 2.51, *p* = .012, *r* = .27) but did not spend significantly more time on the web page than participants in the online condition. Of the prescribed 13 assignments, participants completed a mean number of 9.2 (71%) in the face-to-face condition and 4.2 (32%) in the online condition, a difference that was significant (*Z* = 3.36, *p* < .001, *r* = .37). The mean number of log in occasions, the mean total number of minutes being logged in and the mean number of completed assignments for each condition can be seen in Table 2.

**Table 2:** Descriptive statistics of the outcome variables and statistical differences between the two conditions (*n* = 86)

Measure of adherence	All *M* (*SD*)	Face-to-face *M* (*SD*)	Online *M* (*SD*)	*Z*	*p*	*r*
Log in occasions	4.2 (3.3)	5.0 (3.3)	3.7 (2.0)	2.51	.012	.27
Total time on web page	89.2 (85.0)	91.3 (77.0)	86.4 (95.8)	0.60	.56	.06
Completed assignments	7.6 (4.8)	9.2 (4.1)	4.2 (4.5)	3.36	<.001	.37

### Associations between motivation and adherence

None of the background variables gender, marital status or age was significantly correlated with any of the measures of adherence. In the whole sample, only the SIMS Intrinsic subscale was correlated with total number of log in occasions (*rho* = .27, *p* = .014) and the number of completed assignments (*rho* = .25, *p* = .022). The TCS was correlated only with the number of completed assignments (*rho* = .22, *p* = .048). Analyzing each condition separately yielded only non-significant correlations between the SIMS and the TCS on the one hand and the variables of adherence on the other. Several of the VAS-scales, as well as the VAS-scale index, were significantly correlated with both log in occasions and number of completed assignments. However, when analyzing each condition separately none of the VAS-scales was significantly correlated with adherence in the face-to-face condition and in the online condition, only the Relevance scale was significantly correlated with log in occasions and the VAS-scale index with the number of completed assignments, see Table 3.

**Table 3:** Correlations (Spearman’s *rho*) between the VAS-scales and the outcome variables (*n* = 86)

	Expertise and benevolence	Accountability	Pleasure and mastery	Relevance	Encouragement	Obstacles	Index
All participants							
Log in occasions	.22	.31**	.32**	.28	.34**	-.18	.36**
Total time on webpage	.19	.10	.04	.07	.11	-.12	.07
Completed assignments	.35**	.34**	.34**	.26	.32**	-.17	.37**
Face-to-face condition							
Log in occasions	.09	.13	.14	.05	.24	-.19	.21
Total time on webpage	.22	.09	.02	.16	.03	-.04	.03
Completed assignments	.15	.10	.17	.01	.05	-.07	.13
Online condition							
Log in occasions	.04	.21	.43	.48**	.10	-.16	.34
Total time on webpage	.07	-.25	.10	.27	.10	-.14	.22
Completed assignments	.12	.17	.37	.41	.18	-.21	.55**

Note. ** = *p* < .01

At study end, no participant reported any negative or unintended effects of participating in the study.

## Discussion

The aims of this study were to assess the types of motivation for completing a typical homework assignment and the associations with the subsequent adherence in an experimental psychotherapy setting. A secondary aim was to compare any differences between face-to-face and online interventions in these regards. In line with the study hypotheses, participants reported significantly higher autonomous than externally regulated motivation for the assignment. This is probably a result of the voluntary nature of participating in the study and a sign that the intervention was perceived as meaningful and relevant for participants. The level of adherence in the face-to-face condition was deemed adequate with 94% of participants showing up for the intervention and then completing an average of 71% of the prescribed assignment [3]. Also in line with the study hypotheses, the adherence was considerably lower in the online condition with 78% of participants logging in for the intervention and then completing an average of 32% of the assignments. The difference in dropout prior to the intervention may be due to disappointment with the randomization result, something that was informally suggested by several of the participants but this was unfortunately not measured objectively [32]. It may also indicate that having a face-to-face appointment with a named therapist constitute an informal contract that a vast majority of participants will comply with, in contrast to being asked to log into a web page [23]. While participants in the online condition were informed that an online therapist would guide them on the web page, in hindsight it may have been beneficiary to more specifically appoint participants in the online condition to a named therapist and a specific time for logging in order to minimize drop out. On the other hand, such a procedure would to some degree be incompatible with the common benefits of online therapy, namely a freedom to plan and work with an intervention at a time and pace that suits the individual participant. Future studies may investigate ways to further enhance the initial social contract between participant and therapist, for example by short introductory appointments [48, 49].

Participants in the online condition completed less than half of the number of assignments compared to participants in the face-to-face condition. The results of this study suggest that this may be a result of the lower motivation and intervention credibility reported by participants in the online condition. The low result on these variables implies that an online intervention needs to be very interesting or engaging in order for participants to complete it. However, in previous studies enhancing the presentation of the treatment with media content have not improved the overall adherence to the intervention [50]. The results from the present study are in line with clinical studies which show that adherence is somewhat lower in online compared to face-to-face interventions [38]. There may thus be two different but related processes that lead to dropout in online interventions; a larger proportion of participants drop out before starting the intervention and those who start complete a smaller proportion of the assignments. These different processes may to some extent be explained by the same variables, such as treatment motivation and credibility.

Similar to previous studies, intrinsic motivation (as measured with the SIMS) and intervention credibility were in this study associated with adherence to assignments [51]. The associations could only be seen in the analysis of the whole sample and not in the separate analyses for each condition, arguably because of low statistical power. In contrast, all of the VAS-scales except Obstacles and Relevance were associated with the number of log in occasions and number of completed assignments. The lack of significant associations between the VAS-scales and intervention adherence in the face-to-face condition is difficult to explain. One reason could be the restricted variance in outcome variables in this subgroup. Another reason may be that adherence in face-to-face interventions is associated with completely other variables not measured in the present study. Regardless, the high adherence in the face-to-face condition is probably not caused by a perceived pressure to complete the assignment since neither the SIMS Extrinsic or the Accountability VAS-scale were significantly higher in the face-to-face compared to the online condition. In the online condition, there was a moderate correlation between the Relevance VAS-scale and VAS-scale index on the one hand and adherence to the intervention on the other hand, not seen in the face-to-face condition. The Relevance VAS-scale corresponds to long term goals, or identified motivation. Participants in the online condition who experienced the intervention as meaningful for the long term thus adhered to a higher degree. It is important to remember that several of the VAS-scales showed high intercorrelations and that the specificity of the individual scales could be questioned. However, the VAS-scales index was significantly associated with the three measures of homework adherence which suggests at least a general relevance of these constructs.

Participants in the face-to-face condition reported higher levels on the Intrinsic motivation subscale and lower levels on the Amotivation subscale of the SIMS compared to participants in the online condition. This suggests that it is relatively pleasant to meet with a therapist face-to-face and that receiving psychoeducation online is less intrinsically rewarding for participants. That participants in the face-to-face condition reported lower scores on the Amotivation subscale further suggests that completing the assignments felt overall more important after meeting a therapist than after completing the online psychoeducation. There was also a difference in intervention credibility that indicates that participants in the online condition had more doubt about the plausibility of the assignment, something that has previously been seen is crucial for psychotherapy outcomes [44, 50].

Of the VAS-scales used in this study to investigate the factors associated with assignment adherence identified by Kazantzis [11], participants in the face-to-face condition reported higher levels on the Expertise and benevolence and Encouragement scales compared to participants in the online condition. These results are in line with the results on the SIMS and may be expected given that these two constructs are associated with the relationship between participant and therapist and the limited contact between participants and study staff in the online condition. In contrast, working alliance in full-length guided internet-based psychotherapy is often on par with that of face-to-face psychotherapy, but few direct comparisons have been conducted [52]. Somewhat surprisingly, in this study, the levels on the Accountability subscale was not different between participants in the two conditions which may indicate that all participants expected to be followed up and felt responsible for their assignment to a similar degree. This could be explained by the fact that all participants were informed before the intervention that they would be contacted at the end of the intervention and asked to provide feedback. However, accountability is associated mainly with extrinsic motivation, a type of motivation that has shown to be negatively associated with adherence to assignments [50]. The follow-up procedure in this study was employed in order to mimic the situation in psychotherapy where patients can expect to be asked about assignments on their subsequent appointment. Though the intervention provided in this study did not constitute a treatment, the results from the Relevance scale showed no signs that participants considered the assignment irrelevant. The Pleasure and mastery scale is most closely associated with intrinsic motivation and showed the same pattern as the SIMS Intrinsic motivation subscale with significantly higher levels in the face-to-face condition compared to the online condition. Lastly, participants in the online condition reported a higher degree of obstacles compared to the participants in the face-to-face condition. This may correspond mainly to the technical difficulties that unfortunately still exist when using advanced web applications.

Taken together, the results suggests that while most participants show high levels of adherence to an assignment in a face-to-face intervention, it is primarily people who report high levels of intrinsic and/or identified motivation that will adhere to the assignment in an online intervention. One interpretation of the differences between conditions may be that therapists who meet participants with low motivation face-to-face are able to identify this potential problem and actively work to increase the participant’s motivation, especially if therapists are trained and highly skilled. This could be one of the reasons why participants in the face-to-face condition reported lower levels of amotivation than participants in the online condition. Implementing a similar system in online interventions may be possible but is probably more difficult [26].

This study had several limitations. First, the psychotherapy analog model used in this study has not been previously evaluated and whether the results can be generalized to clinical psychotherapy is uncertain. The psychotherapy model was designed to mimic all major aspects of psychotherapy but the intervention did not constitute a treatment, and the participants were not burdened or help-seeking. However, the Relevance scale mean of 65.3 indicates that at least in general, the participants did not experience the intervention as irrelevant. Also, the results of the present study regarding the importance of intervention motivation, credibility and adherence are in line with the results seen in clinical studies, providing some support for the validity of the model. Psychotherapy analog studies will never replace clinical trials when investigating psychotherapy effects since clinical outcomes such as symptom reduction cannot be investigated but may play a role in explorative research and for generating hypotheses. An alternative strategy may be to conduct longitudinal studies in clinical settings which may provide more ecological but also less distinct results. Second, the sample was recruited among university students and not from a clinical population. Students are in general probably less burdened and more able to engage in homework assignments than many psychiatric patients which could affect the results of this study. In some studies, student samples show symptom levels that are similar to clinical samples but in this study, the mean values were close to those seen in community samples [53]. The sampling strategy was chosen since the intervention was not considered a treatment and it was deemed more ethical to provide it to people who reported interest in changing problematic behavior but without a clinical need. Self-reference recruitment procedures lead to biased samples but are often used in psychotherapy research for practical and ethical reasons. In this study self-reference was deemed adequate since participants curiosity for the intervention to some extent could be viewed as mimicking the need for an intervention seen in help-seeking individuals. However, using a non-clinical sample limits the generalizability of the results and the study should be replicated in clinical populations. Third, the instruments used in this study have not been evaluated in psychotherapy research and their psychometric properties are uncertain in this context. Several of the instruments showed internal consistency values just above .70, which is often used as the lower limit for adequate reliability, and this limits the ability to draw firm conclusions somewhat. The VAS-scales were designed specifically for this study since the Homework Rating Scale II did not fit the psychotherapy model used. The psychometric properties of these VAS-scales are unknown, but the results were never the less significantly associated with the outcome variables, suggesting some validity of the constructs. While some overall conclusions may be suggested, one should be very careful when drawing specific conclusions based on the results from the VAS-scales. In future studies of homework adherence, it may be important to include the HRS II in order to facilitate comparisons between studies and improve generalizability. Fourth, measuring adherence to assignments is difficult since there are several variables and properties to consider, especially in online interventions [32, 54]. The number of completed assignments was chosen as the main outcome variable in the present study. The number of log in occasions and time spent are secondary outcome variables and are arguably less important for treatment outcome. Of these two variables, the number of log in occasions may be preferable, despite the more restricted range, since time spent on the web page is probably less reliable. Another approach could be to focus on and assess the quality of performed homework rather than quantity [55]. The quality of homework is generally more difficult to measure than quantity and may suit some forms of assignments better than others [56]. Fifth, the online procedure for filling out the assignment form is not typical for standard psychotherapy and this may have affected the results. However, the participants in the face-to-face conditions showed a high level of adherence so it seems that the online procedure was not perceived as an insuperable obstacle. Regardless, this procedure may hamper the generalizability of the results. Lastly, while there were significant differences regarding most of the motivation variables between the two conditions, the rather weak associations between motivation and adherence suggests that there are other important unknown variables not measured in this study.

## Conclusions

In conclusion, the results of the present study overall confirm the study hypotheses that participants in traditional face-to-face interventions report higher levels of motivation and adherence to assignments compared to in online interventions. In line with previous studies, adherence to assignments was associated with intrinsic motivation and intervention credibility, especially in the online condition. It thus seems that participating in online interventions puts higher demands on participants’ inherent motivation and belief in the treatment model than in face-to-face interventions. Participants who meet a therapist face-to-face experience intrinsic motivation for an assignment rather than extrinsic pressure. At the same time, participants in the face-to-face condition not only reported higher levels of motivation than participants in the online condition but also higher adherence to the assignment regardless of motivation. This suggests that autonomous motivation is a more important variable in online interventions than in face-to-face interventions. Based on these finding it should be possible to identify patients who report low motivation and are at risk of low treatment adherence or dropout. This study also indicates that adherence can be affected by affecting motivation which should facilitate further experimental research in this area. Future research is needed to investigate how motivation can be increased for participants in online interventions and to explain in more detail variables associated with adherence in face-to-face interventions.

## Abbreviations

DASS: Depression Anxiety Stress Scale
HRS: Homework Rating Scale
SDT: Self-determination theory
SIMS: Situational Motivation Scale
TCS: Treatment Credibility Scale
VAS: Visual Analogue Scale
